# Exploring online search behavior for COVID-19 preventive measures: The Philippine case

**DOI:** 10.1371/journal.pone.0249810

**Published:** 2021-04-08

**Authors:** Adrian Galido, Jerina Jean Ecleo, Atina Husnayain, Emily Chia-Yu Su

**Affiliations:** 1 Department of Information Technology, College of Computer Studies, MSU-Iligan Institute of Technology, Iligan, Philippines; 2 Graduate Institute of Biomedical Informatics, College of Medical Science and Technology, Taipei Medical University, Taipei, Taiwan; 3 Faculty of Medicine, Department of Biostatistics, Epidemiology, and Population Health, Public Health and Nursing, Universitas Gadjah Mada, Yogyakarta, Indonesia; 4 Clinical Big Data Research Center, Taipei Medical University Hospital, Taipei, Taiwan; The University of Hong Kong, CHINA

## Abstract

Public health agencies have suggested nonpharmaceutical interventions to curb the spread of the COVID-19 infections. The study intended to explore the information-seeking behavior and information needs on preventive measures for COVID-19 in the Philippine context. The search interests and related queries for COVID-19 terms and each of the preventive measures for the period from December 31, 2019 to April 6, 2020 were generated from Google Trends. The search terms employed for COVID-19 were coronavirus, ncov, covid-19, covid19 and “covid 19.” The search terms of the preventive measures considered for this study included “community quarantine”, “cough etiquette”, “face mask” or facemask, “hand sanitizer”, handwashing or “hand washing” and “social distancing.” Spearman’s correlation was employed between the new daily COVID-19 cases, COVID-19 terms and the different preventive measures. The relative search volume for the coronavirus disease showed an increase up to the pronouncement of the country’s first case of COVID-19. An uptrend was also evident after the country’s first local transmission was confirmed. A strong positive correlation (r_s_ = .788, p < .001) was observed between the new daily cases and search interests for COVID-19. The search interests for the different measures and the new daily cases were also positively correlated. Similarly, the search interests for the different measures and the COVID-19 terms were all positively correlated. The search interests for “face mask” or facemask, “hand sanitizer” and handwashing or “hand washing” were more correlated with the search interest for COVID-19 than with the number of new daily COVID-19 cases. The search interests for “cough etiquette”, “social distancing” and “community quarantine” were more correlated with the number of new daily COVID-19 cases than with the search interest for COVID-19. The public sought for additional details such as type, directions for proper use, and where to purchase as well as do-it-yourself alternatives for personal protective items. Personal protective or community measures were expected to be accompanied with definitions and guidelines as well as be available in translated versions. Google Trends could be a viable option to monitor and address the information needs of the public during a disease outbreak. Capturing and analyzing the search interests of the public could support the design and timely delivery of appropriate information essential to drive preventive measures during a disease outbreak.

## Introduction

The number of global infections for the coronavirus disease, or COVID-19, has gone past 500,000 after around three months since the onset of the outbreak [1]. The first case of an unknown outbreak, which came to be known as COVID-19, reportedly emerged on December 12, 2019 from the city of Wuhan in China [2] and has since shown transmission across the globe. On March 11, 2020, the World Health Organization (WHO) characterized the outbreak as a pandemic [3].

As of March 28, the number of confirmed cases in the Philippines exceeded 1,000 with the Department of Health (DOH) mentioning that cases are bound to increase as the testing efforts are expanded [4]. From [5], cases of pneumonia cases with unknown etiology were reported in Wuhan, China on December 31. This would later be regarded as an outbreak of the novel coronavirus or COVID-19. The country’s first case of infection was reported on January 30 and its first local transmission was confirmed on March 7 [6]. As a measure to contain the spread of the virus, a State of Public Health Emergency was declared on March 8 with Coronavirus alert Code Red Sublevel 1 through Proclamation No. 922, s.2020. This alert was raised to Code Red Sublevel 2 on March 12. The government also declared the imposition of stringent social distancing measures in the National Capital Region (NCR)—then the country’s most affected region—effective March 15. A memorandum from the Executive Secretary placing the entire Luzon under enhanced community quarantine was declared on March 16, 2020. The entire country was, eventually, placed under a state of calamity due to the COVID-19 threat on March 18.

While efforts to develop pharmaceutical interventions are underway, public health authorities as well as governments among other agencies have been putting in place preventive measures to curb the spread of the virus. Several nonpharmaceutical interventions conveyed by health authorities to help flatten the curve have been circulating in the television as well as online channels. These include social or physical distancing, proper hand washing and community quarantine to list a few.

Disseminating information to the public can be an essential component to reduce further cases. The myriad of social networks provides communities or individuals alike some support for their information needs. Twitter and Facebook have found utility as information sources. Google Trends (GT) is another option which has been notable in disease surveillance. The online search behavior for incidents including disease outbreaks can reflect public interest or, as in [7], be a gauge to measure the public attention.

Google leads the search engine market with over 96% of market share as of December 2018 in the Philippines [8]. Google Trends is a free tool that provides top search or trending queries from Google searches. The tool features the visualization and comparison of the volume of queries over time. Several studies have utilized GT to examine search queries and provide disease surveillance [9–13], i.e., capturing and investigating public interest, for the context of diseases as Ebola, Influenza and HIV among others.

This study aims to explore the online search behavior on preventive measures for COVID-19 in the Philippines. The objectives of the study, more specifically, are to analyze (1) the search interests on COVID-19 and some preventive measures relative to COVID-19 related events; and (2) the relationship between the search interests for COVID-19, some preventive measures and the country’s number of cases. Analyzing the search interests could be useful to gauge the public’s awareness on the relevant preventive measures for the emerging disease outbreak. Analyzing the search interests could also contribute to enabling concerned stakeholders to communicate more effectively to the public.

## Methods

Google Trends provides a query index normalized from the total volume of queries on the specified region over a time period [13]. The query index, or relative search volume (RSV), of 100 suggests that the search term had the most search volume on the instance, i.e., the day, relative to the search volume for the time period being analyzed.

A combination of several terms to generate the search interest for the coronavirus disease was used. Google Trends allows for querying a conceptually similar set of terms through Boolean logic [14]. For this study, the search terms associated with COVID-19 included coronavirus, ncov and covid-19. To account for varying spellings, “covid19” and “covid 19” were also included.

The featured story in Google Trends highlighted measures that individuals searched for to protect themselves from infection including “face mask”, “hand sanitizer” and “hand washing.” Free queries in Google Search were done to identify other preventive measures for COVID-19. Searches were made in English and only results in the said language were considered. To obtain search terms for preventive measures, an initial search on how to “avoid coronavirus”, “coronavirus prevention or protection” and “prevent covid-19” was made. Measures that the public were likely to make additional query on from announcements of public health authorities, such as the Department of Health, and news sources, were consolidated. In addition to the preventive measures from Google Trends’ featured story, “cough etiquette” [15–17], “community quarantine” [18] and “social distancing” [15–17,19] were identified. Further, “social distancing” and “community quarantine” were evident in government mandates as the outbreak progressed.

The final set of search terms were “coronavirus or ncov or covid-19 or covid19 or "covid 19"” for COVID-19 and “community quarantine”, “cough etiquette”, “face mask” or facemask, “hand sanitizer”, handwashing or “hand washing” and “social distancing” for the preventive measures. The set of preventive or protective measures for COVID-19 collected can be grouped into three sets: i) personal protective items (or measures that might need to be purchased), i.e., face mask and hand sanitizer; ii) personal protective practices (or action-oriented measures), i.e., cough etiquette, hand washing and social distancing; and iii) community measures, i.e., community quarantine or enhanced community quarantine, that were mandated by government agencies.

The search interests for each of the preventive measures were generated for the period covering from December 31, 2019 to April 6, 2020 using Google Trends. The 6th of April marked a month after the country’s first local transmission. The data on the related top queries utilized for the study were retrieved on April 13, 2020. Data for the new daily COVID-19 cases were obtained from the repository of the Center for Systems Science and Engineering at Johns Hopkins University [20].

Spearman’s correlation, performed using SPSS, was utilized to determine the relationship between the search terms, including terms associated with COVID-19 and the preventive measures, and the new COVID-19 daily cases.

## Results

### Search interest and timeline of events and related queries

#### COVID-19 terms

**Fig 1:**
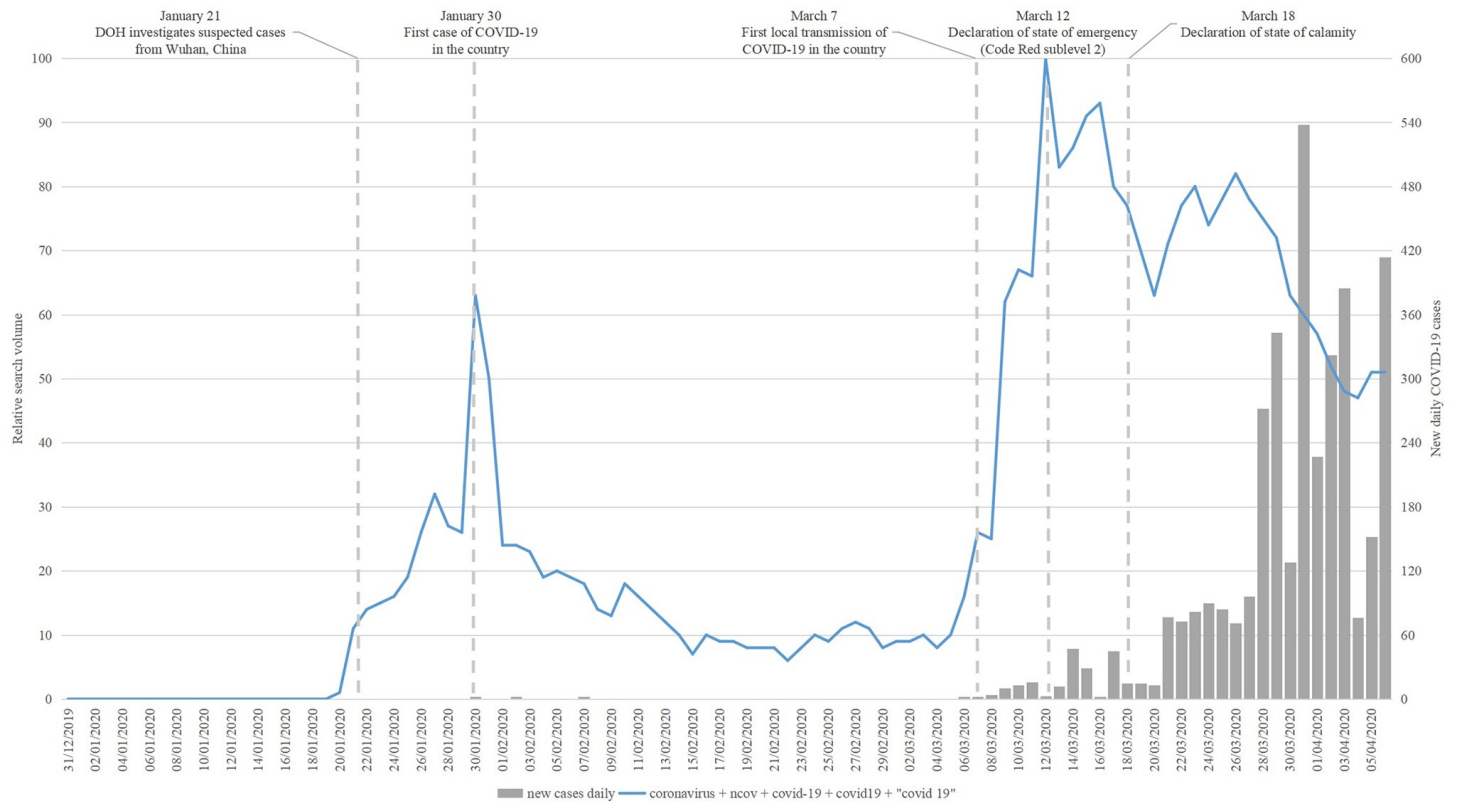
Search interest for terms pertaining to the coronavirus disease for the Philippines.

The public’s interest for coronavirus showed an uptrend (Fig 1) from the middle of January 2020 and eventually reached an initial peak at 63 points on January 30. The initial peak coincided with the date marking the country’s first case of COVID-19. There was a decline, from then, up around early March and a reversal to an uptrend leading to the summit of 100 points on March 12. The pronouncement of the upcoming enforcement of the community quarantine for Metro Manila was also made on the said date. For the study period considered, the related top queries suggest that the public sought online information on the symptoms of the infection and the number of cases in the country (Table 1).

**Table 1:** Related top queries and search interest value for COVID-19 terms.

Related top query	RSV	Related top query	RSV
symptoms	100	italy	19
philippines covid 19	66	ncov philippines	18
philippines coronavirus	62	coronavirus cases	17
coronavirus symptoms	55	philippines covid 19 cases	17
coronavirus update	44	covid 19 news	17
corona	38	china coronavirus	16
covid 19 symptoms	32	covid 19 update philippines	16
covid 19 cases	32	coronavirus news	15
covid 19 update	31	coronavirus philippines update	15
corona virus	29	ncov symptoms	15
symptoms of coronavirus	23	who	13
doh	20	ncov update	12
coronavirus in philippines	19		

#### Personal protective items

**Fig 2:**
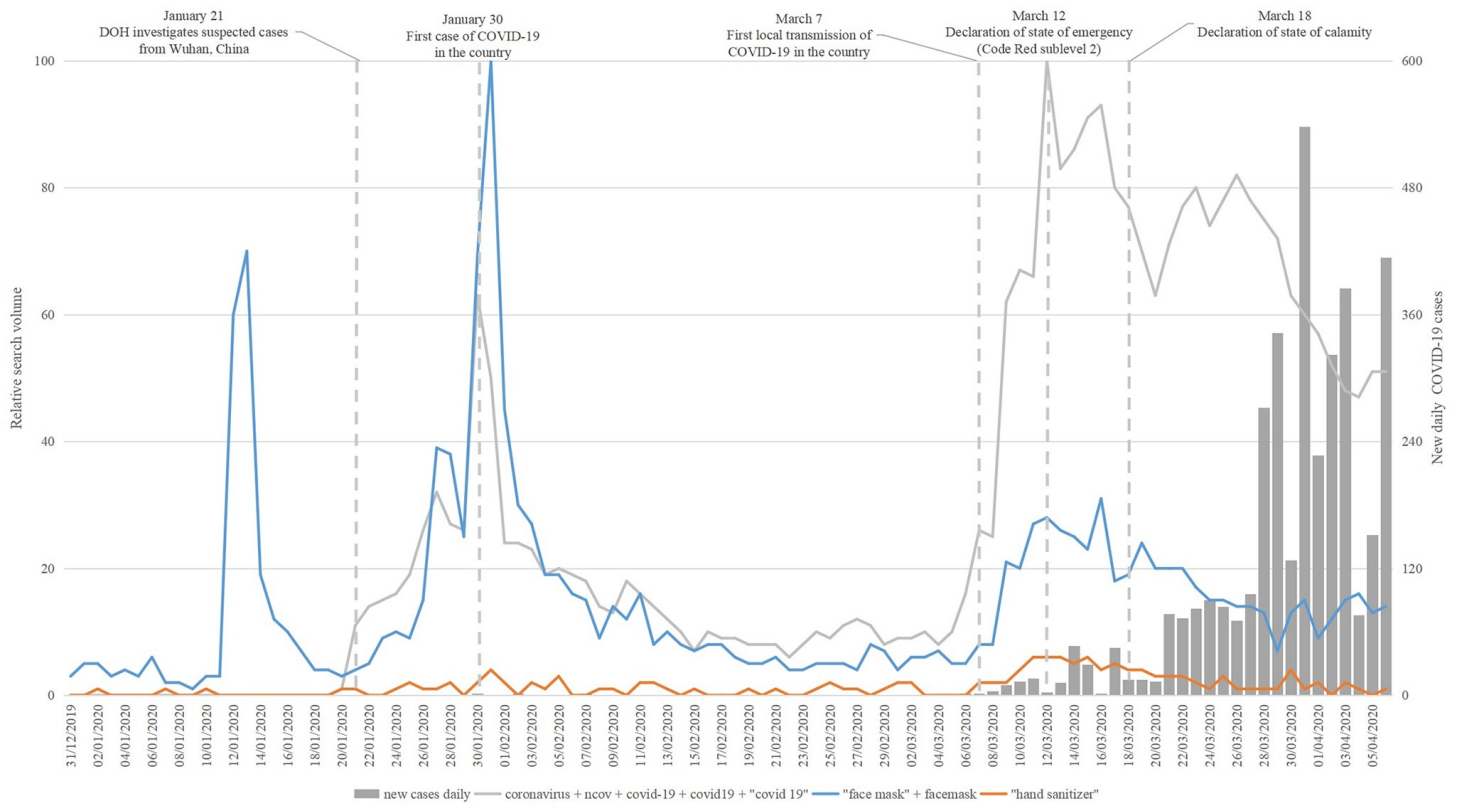
Search interests for personal protective items.

Fig 2 shows the search interests for face mask and hand sanitizer over the study period. Apparently, there were more interests on face mask compared with hand sanitizer. More so, the search interest for face masks increased early on and the latter part of the study period, i.e., January and March, respectively. The related queries for face mask indicated high search interest on the different types of face mask, such as surgical mask or N95 mask, and its proper (Table 2) use while related queries for hand sanitizer indicated high search interest on how to make hand sanitizer (Table 3).

**Table 2:** Related top queries and search interest value for “face mask” + facemask.

Related top query	RSV	Related top query	RSV
face mask use	100	diy face mask	26
surgical mask	86	face mask supplier	26
surgical face mask	84	wearing face mask	25
n95 face mask	73	proper use of face mask	24
n95 mask	69	face mask side	23
face mask philippines	56	face mask for sale	21
how to wear face mask	49	face mask cloth	17
face mask how to use	47	face mask pattern	16
how to use face mask	44	lazada face mask	16
use of face mask	37	washable face mask	15
disposable face mask	33	how to make face mask	15
face mask price	33	indoplas face mask	15
face mask proper use	30		

**Table 3:** Related top queries and search interest value for “hand sanitizer”.

Related top query	RSV
hand sanitizer dispenser	100
how to make hand sanitizer	73
hand sanitizer vs alcohol	40
safeguard hand sanitizer	20

#### Personal protective practices

**Fig 3:**
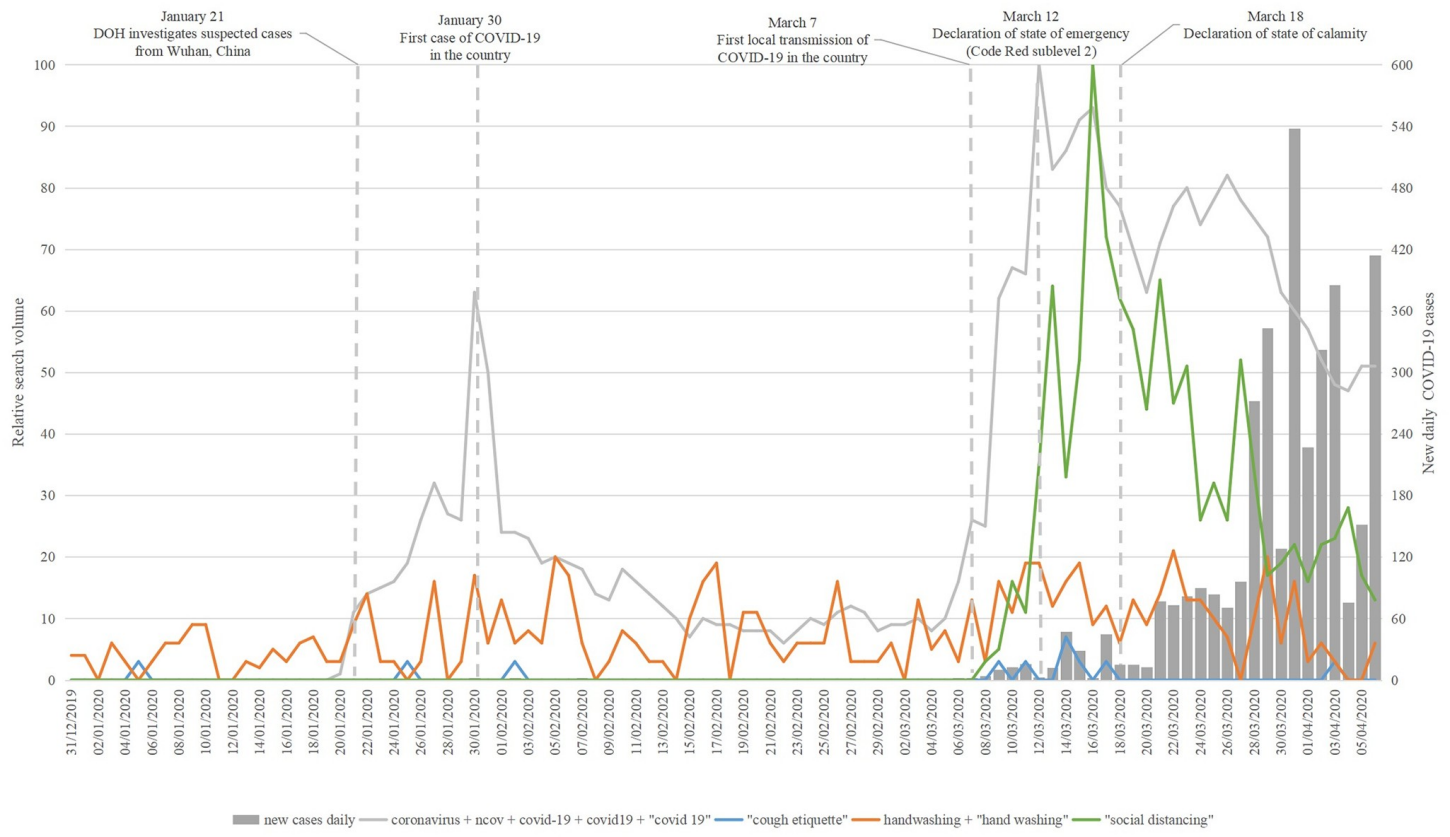
Search interests for personal protective practices.

Fig 3 highlights the search interests for cough etiquette, handwashing and social distancing, i.e., the action-oriented measures. Social distancing had the highest search interest among the three practices. The search interest for handwashing, however, appeared to be more prevalent over the study period. From the related top queries, the public sought information on procedures or guidelines on proper handwashing. There was no result available for the related top queries of cough etiquette. Table 4 lists the top related queries for social distancing.

**Table 4:** Related top queries and search interest value for “social distancing”.

Related top query	RSV
social distancing meaning	100
social distancing philippines	42
social distancing in tagalog	42
social distancing guidelines	30
observe social distancing	24

#### Community measure

**Fig 4:**
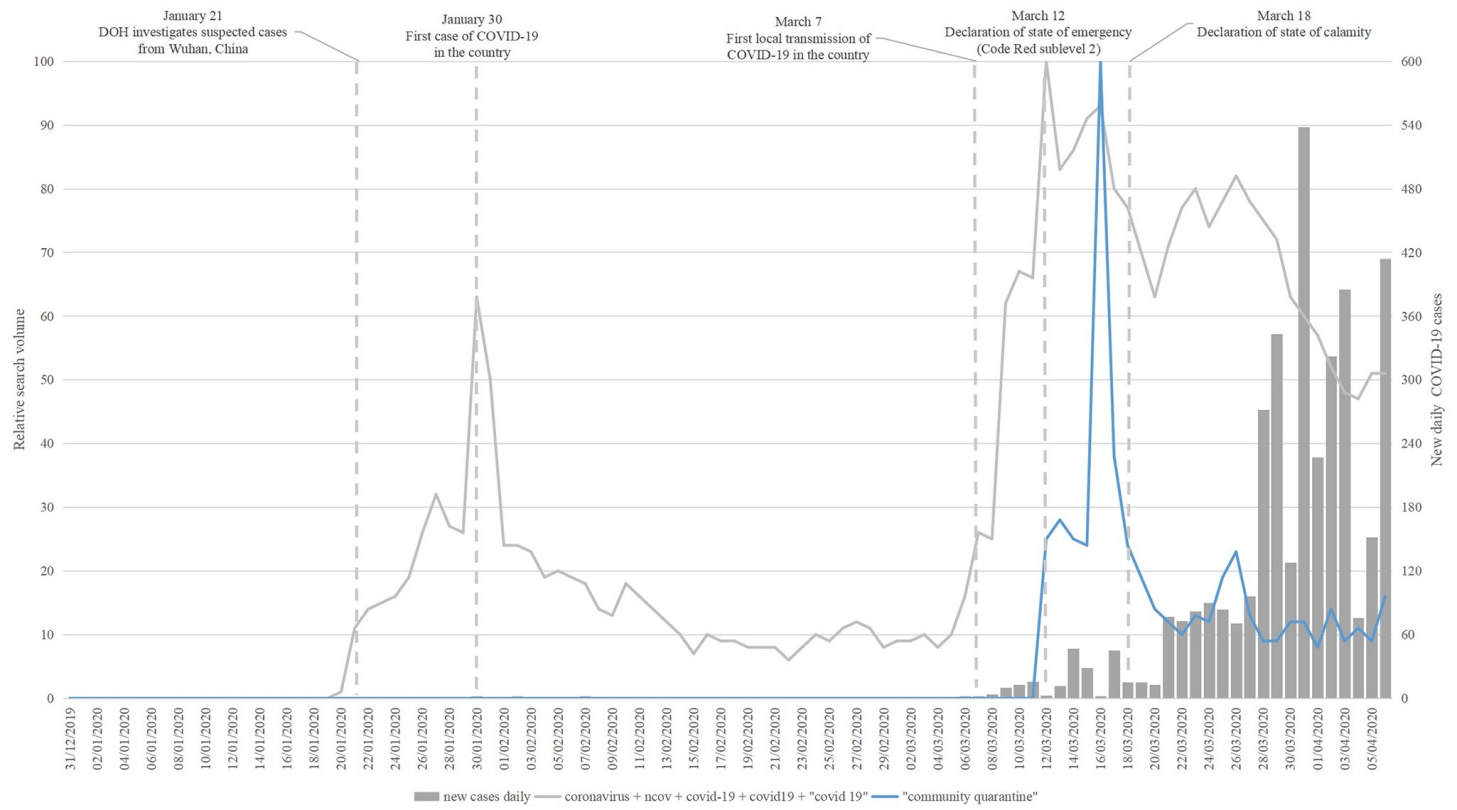
Search interests for community quarantine.

Fig 4 highlights the search interests for community quarantine. The results show a brief decline followed by an abrupt increase in the search interest for “community quarantine” within a week of the announced advance of the country to Code Red Sublevel 2. The said pronouncement incorporates imposing community quarantine in Metro Manila and guidance for local government units in other areas [21]. Table 5 lists the related top queries for community quarantine.

**Table 5:** Related top queries and search interest value for “community quarantine”.

Related top query	RSV	Related top query	RSV
enhanced community quarantine	100	cebu community quarantine	6
community quarantine meaning	34	lockdown meaning	6
enhance community quarantine	31	community quarantine in tagalog	6
community quarantine philippines	31	extended community quarantine	6
community quarantine guidelines	23	enhance community quarantine meaning	4
enhanced community quarantine philippines	15	extreme community quarantine	4
luzon community quarantine	12	community quarantine vs lockdown	4
enhanced community quarantine guidelines	12	guidelines on community quarantine	3
community quarantine tagalog	12	enhanced community quarantine extension	2
what is community quarantine	10	extended enhanced community quarantine	2
enhanced community quarantine luzon	9	enhanced community quarantine in luzon	2
community quarantine metro manila	9	extreme enhanced community quarantine	2
enhanced community quarantine meaning	8		

### Relationship between the new daily COVID-19 cases and the search interests

Table 6 indicates the correlation between the country’s new daily COVID-19 cases and the search interests for the different terms.

**Table 6:** Correlation between new daily cases and a) COVID-19 terms; b) Personal protective items; c) Personal protective practices; and d) Community measure.

		new covid-19 cases (daily)	covid-19 terms
	new covid-19 cases (daily)	-	.788**
a) COVID-19 terms	covid-19 terms	.788**	-
b) Personal protective measures (items)	"face mask" + facemask	.440**	.696**
	"hand sanitizer"	.475**	.693**
c) Personal protective measures (practices)	"cough etiquette"	.249*	.240*
	handwashing + "hand washing"	.305**	.434**
	"social distancing"	.899**	.797**
d) Community measure	"community quarantine"	.843**	.748**

** Correlation is significant at the 0.01 level (2-tailed).

* Correlation is significant at the 0.05 level (2-tailed).

#### Correlation between new daily cases and the search interests

The search interest for the COVID-19 terms and the new daily cases were positively correlated (r_s_ = .788, p < .001). All the search terms for the personal protective measures and for the community measure were positively correlated with the new daily cases. For the personal protective items, the search interest for hand sanitizer (r_s_ = .475, p < .001) was more correlated to new daily COVID-19 cases compared to the search interests for face mask (r_s_ = .440, p < .001). For the personal protective practices, the search interest for social distancing (r_s_ = .866. p < .001) was the most correlated followed by handwashing (r_s_ = .305, p = .002) and, lastly, cough etiquette (r_s_ = .249, p = .013) to the new daily covid-19 cases. The search interest for community quarantine (r_s_ = .843, p < .001) was positively correlated as well.

#### Correlation between search interests for COVID-19 terms and the preventive measures

The search interests for the different measures and the COVID-19 terms were all positively correlated. For the personal protective items, the search interest for face mask (r_s_ = .696, p < .001) was more positively correlated to the search interest for COVID-19 compared to the search interest for hand sanitizer (r_s_ = .693, p < .001). For the personal protective practices, the search interest for social distancing (r_s_ = .797, p < .001) was more correlated compared with the search interest for hand washing (r_s_ = .434, p < .001) and cough etiquette (r_s_ = .240, p = .017). The search interest for community quarantine (r_s_ = .748, p < .001) was positively correlated with the search interest for COVID-19.

## Discussion

The present study intended to explore the online search behavior on the country’s COVID-19 preventive measures. As the country’s new daily cases increased, the public became more aware of COVID-19, social distancing and community quarantine. This finding showed important key information that public health authorities need to provide in order to better communicate with the public.

Several plausible insights could be put forward from the observed information-seeking behavior and information needs of the public relative to COVID-19 related announcements.

Firstly, the search interests of the public demonstrated heterogeneous behavior over the course of the outbreak. Uncertainty can trigger information search ([22] cited in [23]). Information searches tend to be higher at the onset of the outbreak [24]. The decline in internet searches as the outbreak progressed can be attributed to the massive availability of information from alternative channels such as online news reporting, video or radio news reporting, and health expert reporting [25]. Announcements from public health authorities and the media have been noted to influence the health-seeking, i.e., information- or healthcare-seeking behavior of the public during infectious disease outbreaks [26–28]. The study results showed an increase in the public’s interest for COVID-19 from the apparent onset of the outbreak, i.e., a point of uncertainty, up to the pronouncement of the country’s first case, i.e., a point of diminished uncertainty.

Secondly, government information disclosure can direct public attention to focus on the ensuing crises [29]. The vital role of health agencies in disseminating appropriate information during the outbreak cannot be discounted. The Department of Health (DOH), i.e., the country’s national health agency, and the World Health Organization (WHO) were among the related top queries.

Thirdly, the interests for the preventive measures are dissimilar and the information for the measures might need to be tailored. Google Trends can demonstrate utility as a monitoring tool for public restlessness [25]. The perceived availability or otherwise of the preventive measures, such as face mask, can contribute to differing behavior of the search interests over time. A surge in the demand for face (surgical) masks was evident after the country’s first COVID-19 case was confirmed [30].

Tailoring health communication improves information relevance and increases the likelihood of attaining the desired outcomes [31,32]. The protective items—face mask and hand sanitizer—need to be accompanied with information on their type, directions for the proper use or where it can be purchased. The public clamored for product alternatives, specifically do-it-yourself, owing perhaps to the unavailability of the items for purchase. Definitions and guidelines were expected for the protective practices—hand washing and social distancing—and community measures, i.e., community quarantine.

There is an additional observation from the search interest of face mask. The early peak on January 13, 2020 for the search interest on face mask could, apparently, be linked to a different event—increased activity of Taal Volcano. The eventual eruption caused ashfall in several areas in the Calabarzon and neighboring areas [33]. An increase in the purchase demand of face masks, almost instantaneous with the eruption, became evident and resulted to shortages [34,35].

On the relationship between the search interests for COVID-19, identified preventive measures and the new daily cases, the study results suggest that an increase in the number of cases would correspond to an increase search interest for COVID-19. The public became more aware of COVID-19 as the number of new cases increased. For personal protective items, the search interest for hand sanitizer was more correlated with new daily cases while that for face mask was more correlated with the information search COVID-19. Hand sanitizers appeared to have gained more traction, as an alternative means of protection, with the increase of COVID-19 cases. For personal protective practices, the interest for social distancing became apparent with the manifestation of actual COVID-19 cases in the country. A similar observation can be made from the interest for the community measure, i.e., community quarantine. The interests for the said preventive measures increased when the outbreak became more evident.

Using Google Trends has with it accompanying limitations. As highlighted in [36], we acknowledge that the users online might not be representative of the population since all the search data available through Google Trends is anonymized and reflects those with internet access, potentially excluding vulnerable groups or regions where internet uptake could be low. The results of future studies may differ as online search queries might change depending on the terms utilized or the study period considered. Further, the mandatory measures utilized in the study may or may not have been adapted in the locales outside of Metro Manila or Luzon. Additional study might be essential to confirm variations on the information seeking behavior and information needs in the different regions.

## Conclusions

Google Trends could be a viable option for public health authorities as well as other concerned government units to monitor the information-seeking behavior and the information needs of the public. Capturing and analyzing the public’s search interests could support the planning and timely delivery of appropriate information essential to drive preventive measures during a disease outbreak.
